# Analyzing Engagement in a Web-Based Intervention Platform Through Visualizing Log-Data

**DOI:** 10.2196/jmir.3575

**Published:** 2014-11-13

**Authors:** Cecily Morrison, Gavin Doherty

**Affiliations:** ^1^Engineering Design CentreUniversity of CambridgeCambridgeUnited Kingdom; ^2^Microsoft ResearchCambridgeUnited Kingdom; ^3^School of Computer Science and StatisticsTrinity College DublinDublinIreland

**Keywords:** engagement, log-data analysis, data visualisation, Web-based interventions

## Abstract

**Background:**

Engagement has emerged as a significant cross-cutting concern within the development of Web-based interventions. There have been calls to institute a more rigorous approach to the design of Web-based interventions, to increase both the quantity and quality of engagement. One approach would be to use log-data to better understand the process of engagement and patterns of use. However, an important challenge lies in organizing log-data for productive analysis.

**Objective:**

Our aim was to conduct an initial exploration of the use of visualizations of log-data to enhance understanding of engagement with Web-based interventions.

**Methods:**

We applied exploratory sequential data analysis to highlight sequential aspects of the log data, such as time or module number, to provide insights into engagement. After applying a number of processing steps, a range of visualizations were generated from the log-data. We then examined the usefulness of these visualizations for understanding the engagement of individual users and the engagement of cohorts of users. The visualizations created are illustrated with two datasets drawn from studies using the SilverCloud Platform: (1) a small, detailed dataset with interviews (n=19) and (2) a large dataset (n=326) with 44,838 logged events.

**Results:**

We present four exploratory visualizations of user engagement with a Web-based intervention, including Navigation Graph, Stripe Graph, Start–Finish Graph, and Next Action Heat Map. The first represents individual usage and the last three, specific aspects of cohort usage. We provide examples of each with a discussion of salient features.

**Conclusions:**

Log-data analysis through data visualization is an alternative way of exploring user engagement with Web-based interventions, which can yield different insights than more commonly used summative measures. We describe how understanding the process of engagement through visualizations can support the development and evaluation of Web-based interventions. Specifically, we show how visualizations can (1) allow inspection of content or feature usage in a temporal relationship to the overall program at different levels of granularity, (2) detect different patterns of use to consider personalization in the design process, (3) detect usability issues, (4) enable exploratory analysis to support the design of statistical queries to summarize the data, (5) provide new opportunities for real-time evaluation, and (6) examine assumptions about interactivity that underlie many summative measures in this field.

## Introduction

Web-based interventions for improving health have burgeoned over the past 10 years as researchers aim to harness the reach and cost-effectiveness that the Internet promises [1-6]. However, obstinately high rates of attrition have kept them from reaching their potential [7,8]. As the corpus of research studies grows, there have been calls to develop a more scientific approach to the design and evaluation of Web-based interventions: a methodology that includes the analysis of engagement. One important aspect of such a methodology would be to support a more nuanced understanding of how users engage with Web-based interventions, or a science of engagement [9-11].

Previous research has focused on the summative measurement of engagement, such as level of adherence [12,13] or intervention exposure [14-16]. Researchers have also identified factors that correlate with adherence, including patient characteristics [17] (eg, gender), the context of delivery [18] (eg, classroom), aspects of the delivery [19] (eg, therapist support), and characteristics of the intervention itself [20] (eg, tailored content). Attempts to focus more directly on interactive technological elements of an intervention that may facilitate engagement have reported positive correlations between interactive feature inclusion and outcomes [21-24].

Such summative measures do not capture the temporal elements of engagement needed to provide insight into the design of Web-based interventions. This is echoed in recent work that has demonstrated that there is not a linear relationship between usage (either adherence or exposure) and outcome [25]. Rather, a substantial amount of variance seen in adherence between studies can be explained by the characteristics of the Web-based intervention, that is, interactive technological elements [26]. These two findings taken together suggest that it is essential to understand the temporal process of engagement or patterns of use.

Log-data analysis, similar to that used in the analysis of websites in other domains [27], provides a way to capture patterns of use. Log-data has been used, for example, to illustrate changes in frequency of feature usage over time (eg, feedback messages) as well as staged feature usage (eg, starting a mindfulness exercise, but not downloading it) [28]. These authors propose further work on how content/feature integration and intra-usability of features can enable engagement. Such work raises the question of how to capture patterns of use in a way that defines engagement as more than a sum of individual content or feature element usage.

The challenge of using log-data to understand the process of engagement lies in organizing it for productive analysis. In this paper, we present a set of visualizations that capture the process of engagement for individuals and cohorts. The aim of the paper is to stimulate a discussion on ways that log-data can be used to understand user engagement for the explicit purpose of Web-based intervention design.

## Methods

### Data Visualization: Information Visualization

Information visualization can be defined as “the use of computer-supported, interactive, visual representations of abstract data to amplify cognition” [29]. Information visualization can be used either to explore or communicate a set of data [30]. We apply exploratory sequential data analysis [31] to highlight sequential (or temporal) aspects of the data, such as time or module number, that can provide insights into engagement. While this approach has been applied to log-data analysis for session events grouped into a small number of distinct categories [32], there are no precedents for representation of continuous data.

When designing visualizations, it can be helpful to consider Schneiderman’s information seeking mantra: “Overview first, zoom and filter, then details-on-demand” [33]. This can be applied to a single visualization or to linking a series of visualizations. For example, a visualization that depicts navigation paths for an individual could show an overview of all sessions, enable zooming on a particular session, or selecting details of a specific event, or user action. Alternatively, a visualization of usage over time for an entire cohort could support filtering (eg, by gender or initial depression score), and details-on-demand linking to a visualization of an individual’s temporal navigation patterns.

### Data

Data used in the visualizations are drawn from two studies using the SilverCloud platform, which is described below. The first dataset is from a small pilot trial of the SilverCloud platform in a primary-care mental health setting in the United Kingdom [34]. Outcome data, log-data, and interview data were captured for 19 participants. The combination of data allows us to explore engagement patterns of individuals in depth. To complement this small, detailed dataset, we used a larger dataset drawn from the usage of the same intervention in a university setting [35]. The second dataset contains 326 participants, and over 44,838 logged events, which allows us to explore the issues surrounding visualization of a larger cohort.

### Data Transformation Steps

Exploratory sequential data analysis involves the successive transformation of raw data sequences until the product enables statements to be made that answer research or design questions [31]. In this case, the original log-dataset included user ID, time-stamp, and page URL (uniform resource locator) of every action completed, such as reading a content page, saving a journal entry, or updating an activity. The first data processing step was to transform the URLs into meaningful labels. Content page URLs were assigned labels that indicated sequential order. For example, a URL ending with “content/9/54/93/” can be mapped to “Module 2 Subsection 1” or more briefly “2.1”. URLs for features, such as “Journal” or “Mindfulness”, were renamed as such.

The next data processing step is to identify sessions, defined as repeated interaction with the system over an interval of time. To avoid counting periods when the user was not actively engaged with the system, any period of inactivity longer than a threshold starts the count on a new session. The threshold is configurable, and for the examples presented here, a value of 60 minutes is used. The threshold has been set to make allowance for users watching videos repeatedly or taking time to compose longer textual entries for certain exercises. As session time is not a feature in these visualizations, the impact is only in the segmentation of the data into different sessions. A shorter threshold may be appropriate for some interventions or for visualizations where session time is the focus to avoid overestimation of session time.

Each of the individual visualizations entail further transformations of this data, building on these first two steps. These included extracting usage days and first and last content-viewing events within each session for example.

### Visualization Tools

The programs used to produce the visualizations in this paper were developed in the *processing* language and have been made freely available. Applying these visualizations to another dataset requires two elements: (1) a data file that includes user ID, time-stamp, and event identifier (eg, URL), for all actions, and (2) a key file that maps event identifiers to meaningful labels and their intended sequential use, and differentiates between content and other features. The event identifiers will commonly be in the form of URLs, but other formats might be used. Identifiers do not need to be unique—several URLs might map to the same piece of content.

**Figure 1:**
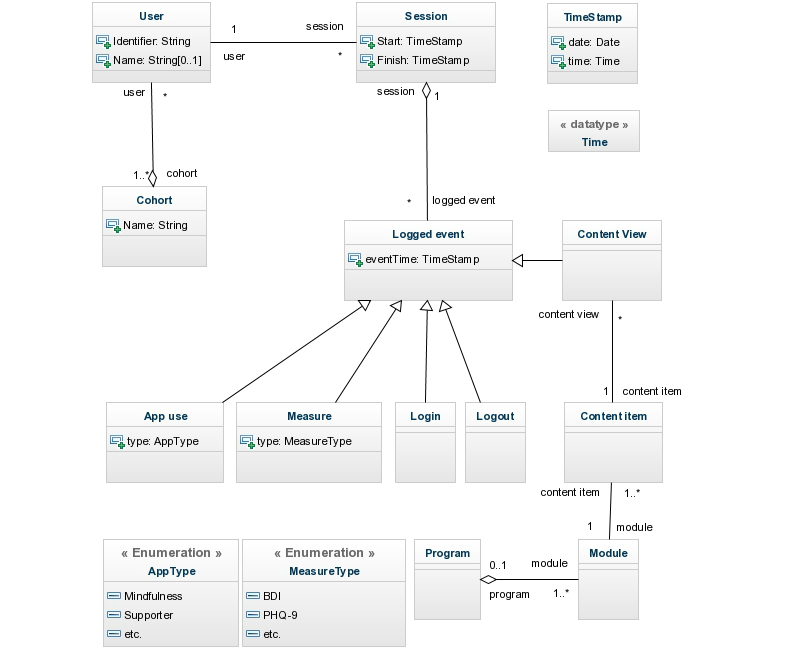
Data model for interactive Web-based interventions.

One program processes the data and outputs a number of spreadsheets that can be checked for correctness as detailed in the readme file. The other programs produce the visualizations presented below. Figure 1 is the data model underlying the visualizations considered in this paper. The full code and a small amount of test data are available in Multimedia Appendix 1.

### Web-Based Intervention: The SilverCloud Platform

The data used to develop these visualizations are drawn from studies that used the SilverCloud platform. SilverCloud is a media-rich, Web 2.0 platform that can be used to quickly build interactive Web-based interventions for common mental health problems. It is specifically designed to improve engagement through the following design strategies:

- Personalization: Users are encouraged to draw together all strands of the intervention and build their own plan or “toolbox” for staying well and managing current and future mood difficulties.
- Interactive exercises: Users can engage with the range of media, such as interactive quizzes, video presentation, Web-based exercises and activities, homework, and mobile diary-keeping. These are meant to encourage reflection and personalization of the information offered in the intervention.
- Guidance and support: Though mainly self-directed, each user in the program is assigned a supporter who provides feedback at specified intervals throughout the intervention on the activities that the user has chosen to share.
- Social features: Users can gain a sense of other people using the system by seeing how many people liked an activity, or by sharing answers to an activity that are visible to all after moderation.

Each module is structured in an identical way and incorporates introductory quizzes, videos, informational content, interactive activities, as well as homework suggestions and summaries. In addition, personal stories and accounts from other clients are incorporated into the presentation of the material.

SilverCloud is an interesting source of data as there are a variety of types of user interaction with the system to explore. As a platform, the focus is on the technology elements (design strategies) that structure the intervention, rather than the content. The sample platform can be used to deliver a range of programs (eg, depression [36] and anxiety [37]). A more detailed description of the implementation for depression (MindBalance) is available in Sharry et al [35], including a video overview.

### Focus of Visualizations

There are three aspects of the SilverCloud platform that feature prominently in the visualizations we discuss in this paper. First, user navigation possibilities are multiple. It is possible to take a linear approach, clicking the forward arrow to get to the next page or activity planned by the content designer. It is also possible to choose one’s own path through the intervention, selecting which module and submodule to start with. The latter approach is similar to navigating a webpage.

Second, apps are used to support the engagement with content. These include an interactive journal, recordings of mindfulness activities, as well as interactive activities, such as creating a “Thoughts, Feelings, Behaviors” cycle. Some of the apps contain material that can be downloaded and used offline. Third, support is provided by weekly reviews. The client’s supporter, generally a health professional, reviews the content that the client has shared and provides encouragement and guidance. A supporter might recognize and articulate the efforts a client has made, or suggest a particular content page or activity.

**Figure 2:**
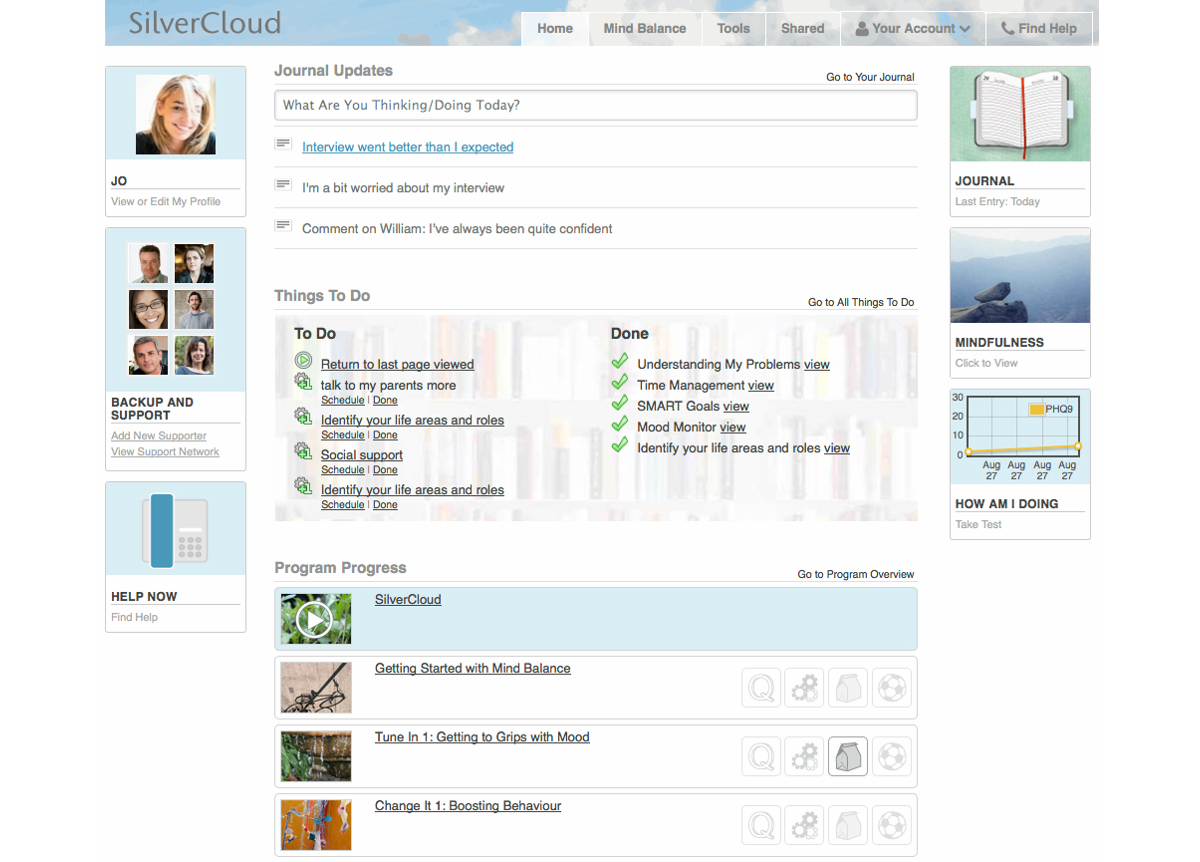
SilverCloud Home Screen, which enables different types of user navigation.

**Figure 3:**
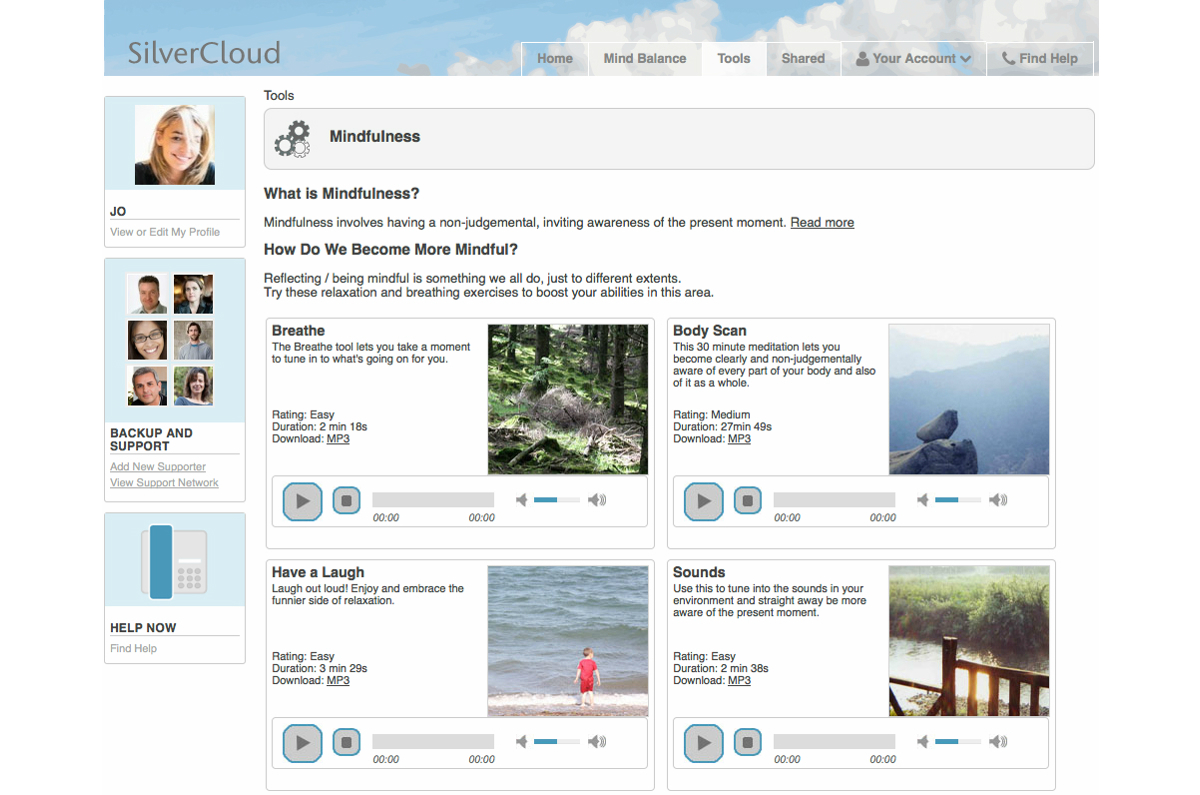
Mindfulness app in SilverCloud.

**Figure 4:**
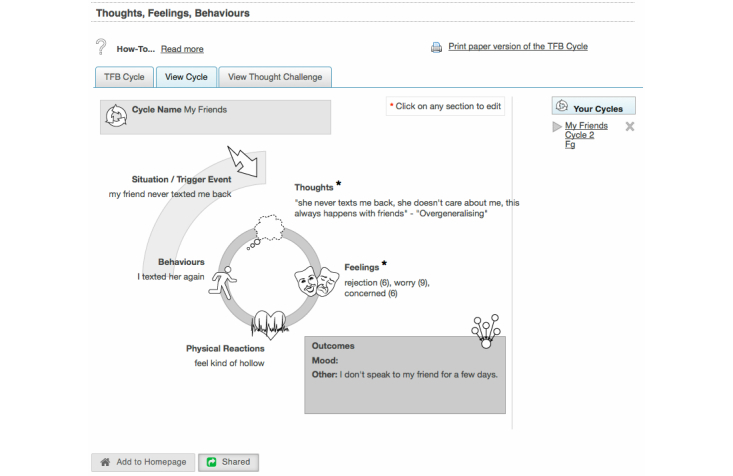
Screenshot of an activity that can be shared for review.

Figures 2-4 illustrate the three main aspects of the user interaction with SilverCloud just described that will be discussed in the example visualizations: (1) user navigation, (2) apps, and (3) reviews.

## Results

### Overview

Four visualizations are presented in this section. The first, Navigation Graph Visualization, illustrates the temporal process of engagement by an individual. The second, third, and fourth visualizations aggregate specific aspects of this first one over an entire cohort. Stripe Graph Visualization illustrates the temporal pattern of use over the intervention period for individual users but is compact enough to be stacked, allowing usage across a cohort to be examined and compared. Start–Finish Visualization depicts the shape of users’ sessions across a cohort, showing the starting and finishing content page of each session. Finally, Next Action Heat Map Visualization aggregates the navigation path through the intervention.

### Navigation Graph Visualization

#### Description

**Figure 5:**
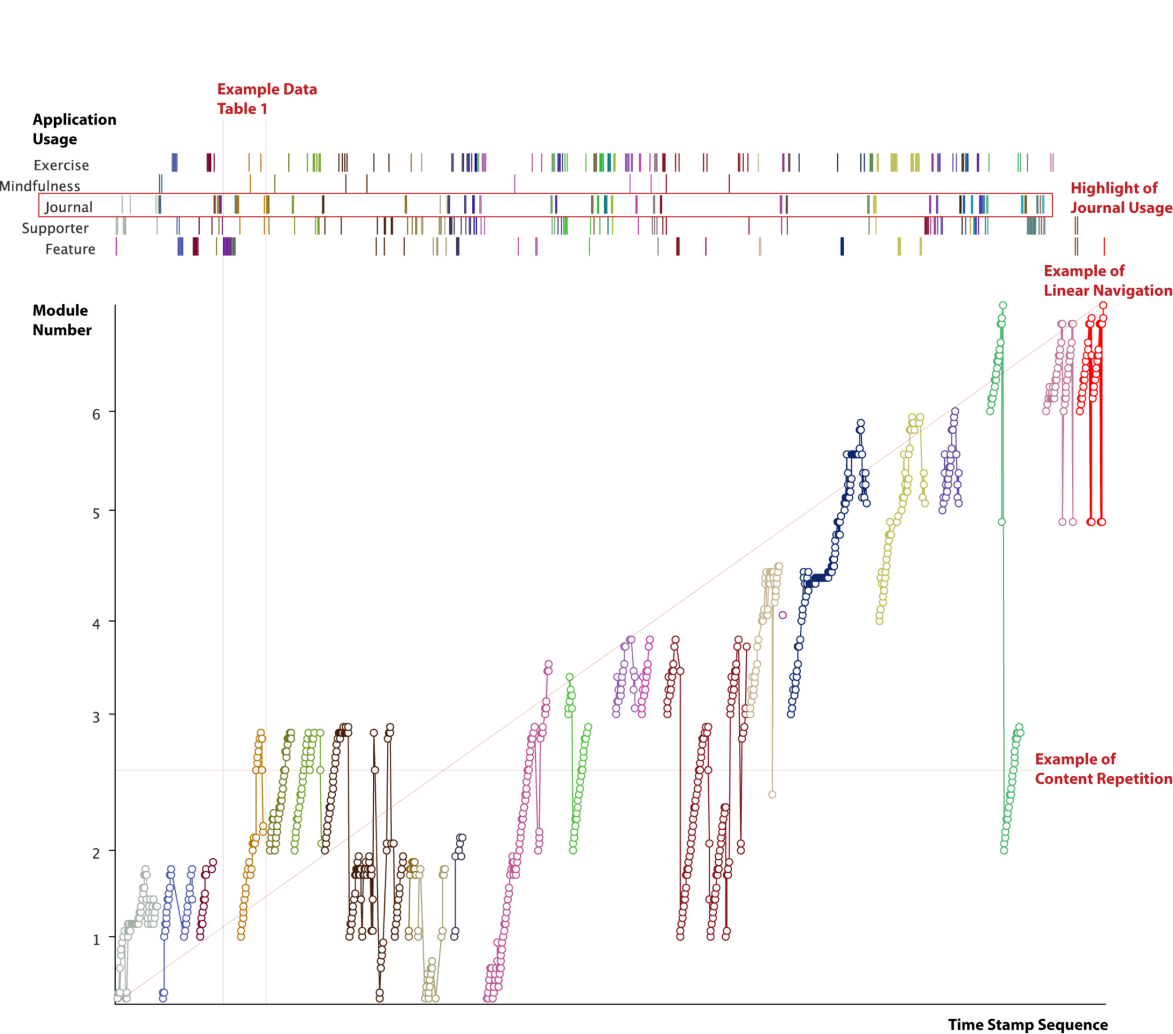
Navigation Graph Visualization showing pattern of use trajectory of a user through SilverCloud: (1) graph section that relates to example data in Table 1, (2) example of content repetition within and between sessions, (3) example of linear navigation, (4) highlighted journal usage.

**Figure 6:**
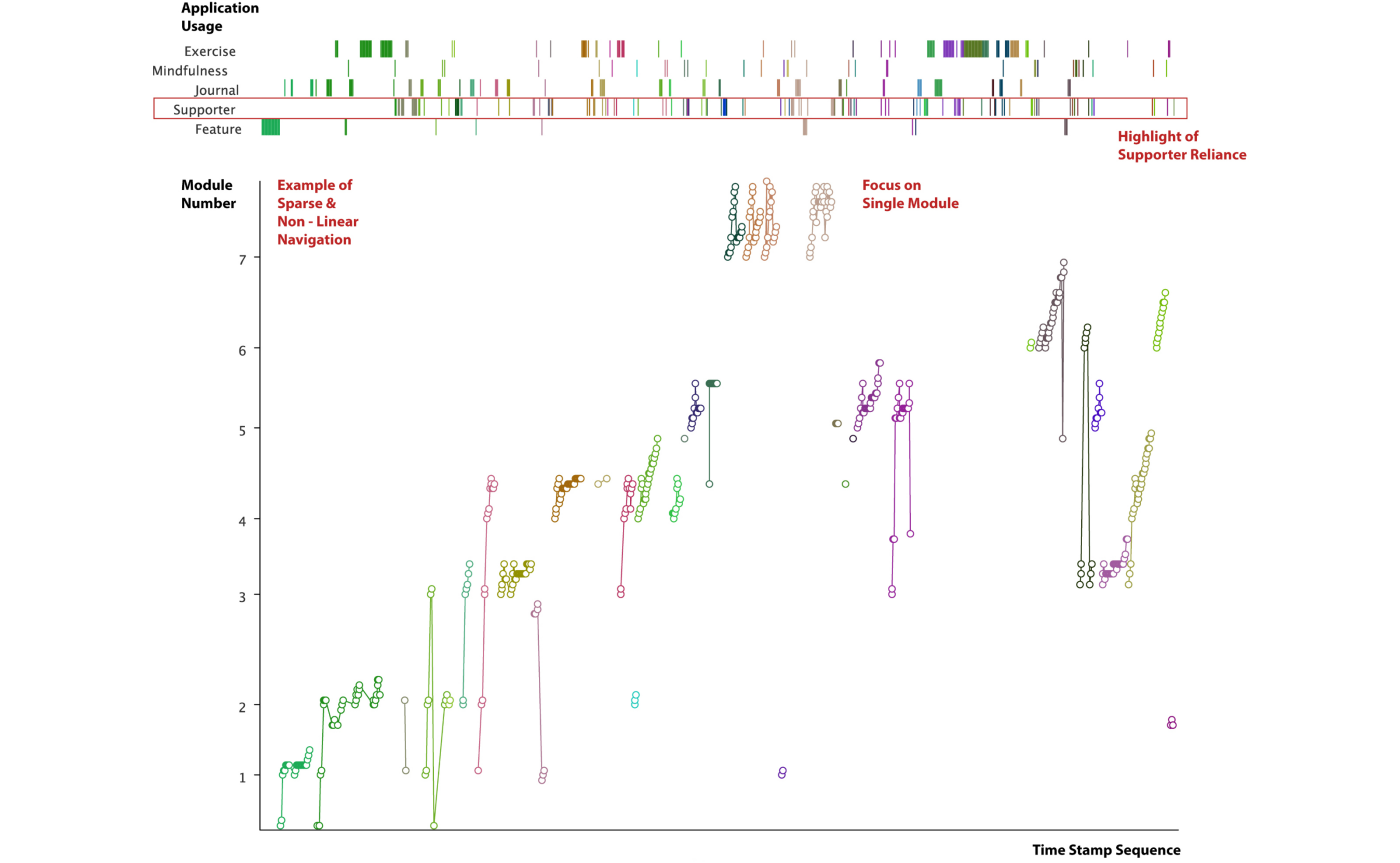
Navigation Graph Visualization showing pattern of use trajectory of a user through SilverCloud: (1) focus on a single module, (2) example of sparse and non-linear navigation, (3) highlight of supporter reliance.

**Figure 7:**
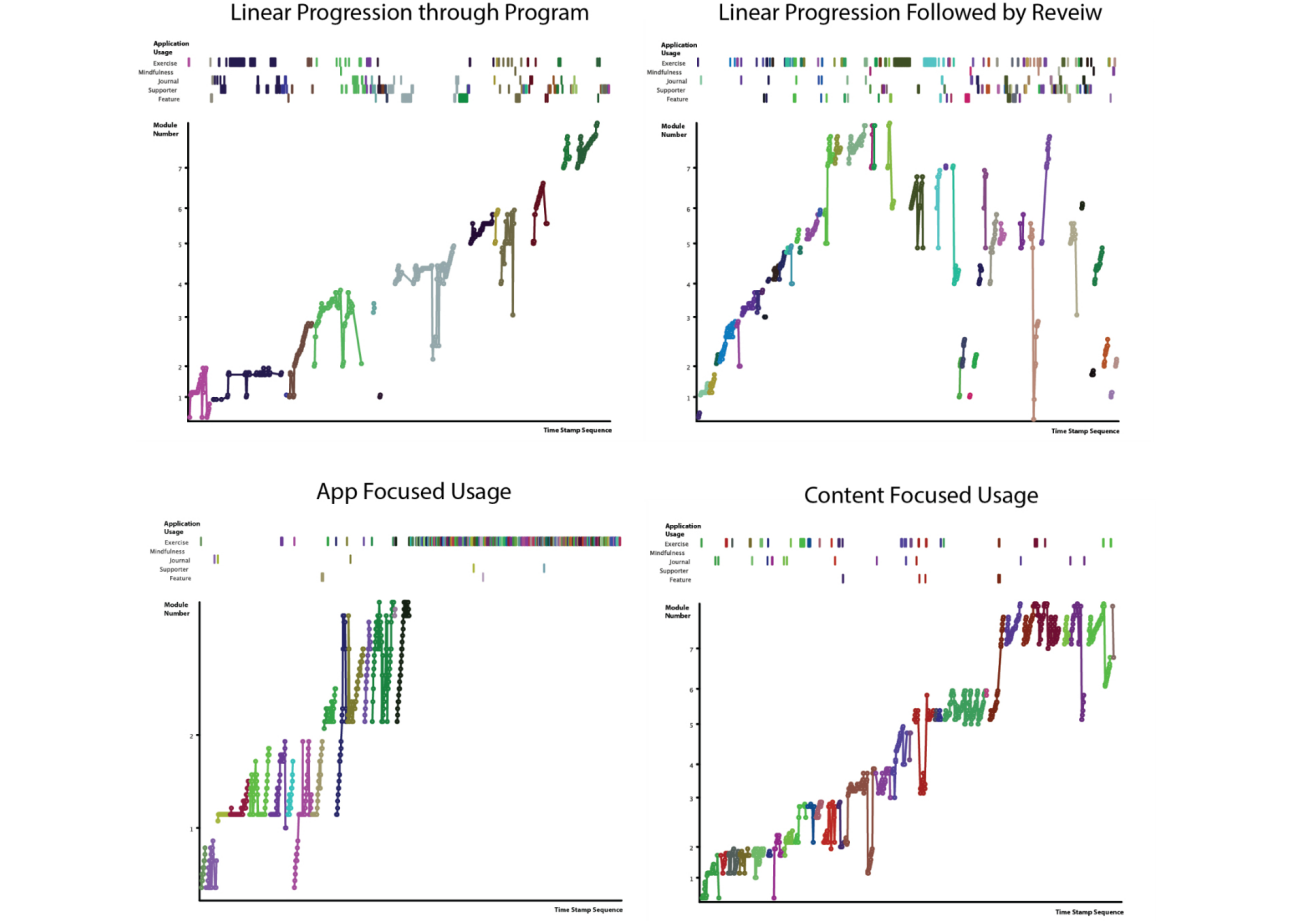
Navigation Graphs Visualization showing 4 different pattern of use trajectories: the top two highlight linear progression versus exploration and review, and the bottom two contrast an app focus to a content focus.

The Navigation Graph Visualization in Figures 5-7 depicts an individual’s temporal process of engagement with the content and interactive features embedded in apps. It consists of two parts: a line graph of content usage and a stripe graph of application usage across the top. The interactive version includes a vertical bar cursor that enables one to line up events between the line and stripe graphs. The horizontal axis shows each content page viewed or app used in sequential order. Sessions are demarcated by space and color. Each content page is marked by module number followed by content page number. For example, 4.3 is the third content page in module 4. Across the top are five categories of apps in the SilverCloud platform: In-content exercise, Mindfulness applications, Journal usage, Review provided by a supporter, and Other interactive features (eg, quizzes). These are temporally sequenced with content usage. The data are taken from both datasets. Table 1 provides a sample of the log-data used to generate the visualization.

The Navigation visualization captures a substantial amount of information about an individual’s pattern of use. At a glance, it is possible to see (1) range of content viewed in a session, (2) repetition of content in a session and across sessions, (3) linearity of content viewed within and across sessions, (4) app usage over time, and (5) interspersal of apps and content.

**Table 1:** Log-data from one session of user Janet, as marked in Figure 1.

Raw log-data			Generated data	
ID	Date and time	URL (website address omitted for brevity)	Sequence assignment	Session number
8	08/03/2012 12:47		Home	4
8	08/03/2012 12:47	bns/main/	Feature	4
8	08/03/2012 12:47	bns/1239/updateItem/	Feature	4
8	08/03/2012 12:47	bns/1230/updateItem/	Feature	4
8	08/03/2012 12:47	bns/1230/updateItem/	Feature	4
8	08/03/2012 12:47	bns/1228/updateItem/	Feature	4
8	08/03/2012 12:47	bns/1241/updateItem/	Feature	4
8	08/03/2012 12:48	journal/add/	Journal	4
8	08/03/2012 12:48	journal/	Journal	4
8	08/03/2012 12:49	journal/1537/delete/	Journal	4
8	08/03/2012 16:41		Home	4
8	08/03/2012 16:41	journal/	Journal	4
8	08/03/2012 16:47	journal/add/	Journal	4
8	08/03/2012 16:47	journal/	Journal	4
8	08/03/2012 16:47	apps/share	Supporter	4
8	08/03/2012 16:47		Home	4
8	08/03/2012 17:18	content/9/53/238/	1	4
8	08/03/2012 17:18	apps/40/i	1.0.1	4
8	08/03/2012 17:18	content/9/53/92/	1.1.0	4
8	08/03/2012 17:18	92-mind-balance-basics/#carousel11	1.1.0	4
8	08/03/2012 17:18	92-mind-balance-basics/#content2	1.1.1	4
8	08/03/2012 17:18	92-mind-balance-basics/#content3	1.1.2	4
8	08/03/2012 17:18	92-mind-balance-basics/#content4	1.1.3	4
8	08/03/2012 17:18	/content/9/53/98/	1.2.0	4
8	08/03/2012 17:18	98-personal-stories/#carousel1	1.2.0	4
8	08/03/2012 17:18	98-personal-stories/#carousel2	1.2.1	4
8	08/03/2012 17:18	98-personal-stories/#carousel3	1.2.2	4
8	08/03/2012 17:18	content/9/53/104/	1.3.0	4
8	08/03/2012 17:18	apps/42/i	1.3.0	4
8	08/03/2012 17:18	apps/41/i	1.3.1	4
8	08/03/2012 17:18	104-activity/#subconcept2	1.3.1	4
8	08/03/2012 17:20	apps/41/addItem	Exercise	4
8	08/03/2012 17:20	104-activity/#subconcept3	1.3.2	4
8	08/03/2012 17:21	apps/14/	Mindfulness	4
8	08/03/2012 17:21	apps/42/i	1.3.0	4
8	08/03/2012 17:21	apps/41/i	1.3.1	4
8	08/03/2012 17:21	104-activity/#subconcept2	1.3.1	4
8	08/03/2012 17:22	content/9/54/239/	2	4
8	08/03/2012 17:22	apps/33/i	2.0.1	4
8	08/03/2012 17:24	content/9/54/93/	2.1.0	4
8	08/03/2012 17:24	apps/33/i	2.0.1	4
8	08/03/2012 17:25	apps/33/i	2.0.1	4
8	08/03/2012 17:27	apps/33/update	2.0.1	4
8	08/03/2012 17:27	content/9/54/93/	2.1.0	4
8	08/03/2012 17:27	content/9/54/99/	2.2.0	4
8	08/03/2012 17:27	content/27-tune-1/99-personal-stories/#carousel1	2.2.0	4
8	08/03/2012 17:30	content/27-tune-1/99-personal-stories/#carousel2	2.2.1	4
8	08/03/2012 17:30	content/27-tune-1/99-personal-stories/#carousel3	2.2.2	4
8	08/03/2012 17:30	content/27-tune-1/99-personal-stories/#carousel4	2.2.3	4
8	08/03/2012 17:31	content/27-tune-1/99-personal-stories/#carousel5	2.2.4	4
8	08/03/2012 17:31	content/9/54/105/	2.3.0	4
8	08/03/2012 17:31	content/9/54/105/	2.3.0	4
8	08/03/2012 17:31	apps/15/i	Exercise	4
8	08/03/2012 17:31	apps/2/i	2.3.1	4
8	08/03/2012 17:31	content/27-tune-1/105-activity/#subconcept1	2.3.0	4
8	08/03/2012 17:31	content/27-tune-1/99-personal-stories/#carousel1	2.2.0	4
8	08/03/2012 17:33	93-getting-grips-mood/#subconcept2	2.1.1	4
8	08/03/2012 17:35	93-getting-grips-mood/#subconcept3	2.1.2	4
8	08/03/2012 18:16	journal/add/	Journal	4
8	08/03/2012 18:16	apps/share	Supporter	4
8	08/03/2012 18:17		Home	4
8	08/03/2012 18:46		Home	4
8	08/03/2012 18:46	journal/	Journal	4

#### Examples

Figures 5 and 6 show the Navigation Graph Visualization from Janet and Robert, respectively. Janet and Robert are two persistent users who had more than 58 sessions each. Both reported substantial benefit from using the intervention. Comparing the visualizations immediately shows that these two users had very different patterns of use.

Janet viewed all of the content pages in a module. We can also see that she repeated content both within a session, as in session 2, and across sessions, as with 2, 3, 4, 7, 10, and 14. Otherwise, Janet took a relatively linear path through the content, looking at the next recommended page. Looking at app usage, the Journal was used at the beginning or end of most sessions. Janet also used the Mindfulness apps and read the Reviews by her supporter.

Robert, on the other hand, had a very different pattern of use. App usage was more prevalent than content usage. The latter was sparse and jumped around across modules. There was, however, substantial focus on the Core Beliefs module, which was done four times in detail. The number of times a Review was viewed (n=28) is striking as only 8 reviews were written.

The most interesting aspect of these visualizations is their diversity. Janet progressed in a more or less linear manner through the content, while Robert jumped around. Janet focused on content and Robert on apps. This difference emphasizes that not all users move through the content in the linear order planned but find their own pathways when allowed. This difference can be seen in a more extreme fashion in the 4 users portrayed in Figure 7.

These visualizations provide an interesting contrast with feedback garnered from interviews. Janet described her use of the application as reading every page but never looking back. Although the log-data indicate that she does indeed read every page, she also repeated a substantial amount of material. This would not be obvious without the visualization. Robert, in contrast, found it difficult to concentrate and said that he clicked around until he found something that he could relate to. Again, although this is indeed true, the log-data indicate that content in all modules was viewed. These examples indicate that people’s usage may not be as straightforward as they describe.

There are some perspectives that the visualization cannot capture. Without interviewing users, we would not know that Janet treated the intervention like a course to be completed and Robert saw it as his sanctuary to help him through sleepless nights. This explains their different patterns of engagement with the content. A further issue is that some of the apps, such as the Mindfulness exercises, could be run in the browser or downloaded. Robert reported listening to these exercises daily from downloaded versions. We cannot capture such elements of intervention use in the log-data.

The visualizations in Figure 7 make apparent very different pattern of use trajectories that would be hard to detect through manual inspection of the data. From these extreme cases, we can tease out important dimensions of patterns of use. The top two examples illustrate that content review is an important element of usage. In the left example, review is done per session, and in the right example, it is done after all the content has been viewed. These examples stand in contrast to enforced linear use in many Web-based interventions and suggests that some summative measures, such as module completion, may not always be appropriate.

The bottom two graphs of Figure 7, in conjunction with Figures 5 and 6, give us more insight into how people interweave content and interactive feature usage (referred to as intra-usability of features [28]). In Figure 7, we can see extreme cases of app-focused and content-focused usage showing the importance of preference in how people engage. Figures 5 and 6 also show these contrasting preferences to a lesser extreme with clear routines emerging quickly. Janet consistently used the diary at the beginning or ends of most sessions for example. By looking at the data presented in the visualization, we get a fuller picture of engagement throughout the application.

These examples draw attention to avenues for future design as well as assumptions commonly made in the evaluation of Web-based interventions. For example, knowing that people return repeatedly to content they find relevant to them, we might look at ways of “refreshing” this content, by presenting new examples or personal stories. When evaluating interactivity, these graphs can help move beyond the idea of isolated feature usage. We could, for example, look at where in the session that features are being used to see if there are patterns that encourage either the quantity or perhaps quality of engagement.

### Stripe Graph Visualization

#### Description

**Figure 8:**
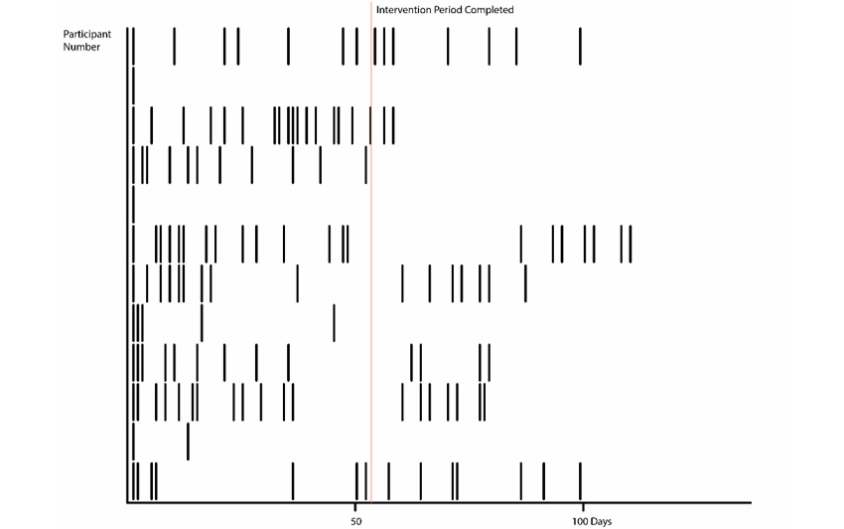
Stripe Graph Visualization showing temporal representation of intervention use by individuals in a cohort (y-axis) over days in the treatment period (x-axis).

The Stripe Graph Visualization in Figure 8 isolates temporal use of the Web-based intervention over the treatment period and makes it comparable in a cohort of users. The horizontal axis is days, and the users are stacked on the vertical axis. A bar is placed on each day of the treatment period in which the intervention is used. The data used to generate this graph are participant ID and date. The data are excerpted from the large dataset.

This visualization captures (1) length of use and (2) consistency of use.

#### Example

The example in Figure 8 shows 12 users. What is most striking about the visualization is that many people used the intervention in bursts sometimes with long periods in between. This is a different pattern of usage than the consistency assumed in most adherence metrics. From a design perspective, this prompts consideration of the engagement goal—is it to make users come back each week or is it to ensure that each burst leaves the user with something to do or think about that is likely to lead to changes in behavior?

It is also interesting to note the significant level of usage beyond the formally supported 8-week period. Most studies tend to focus on the formally defined usage period associated with their post-intervention outcome measures. This suggests that other ways of collecting post-intervention data may be relevant. The visualization is also interesting from a design perspective, suggesting that there may be value in exploring how people use SilverCloud without online guidance after the formal intervention period, in order to provide appropriate support.

### Start–Finish Graph Visualization

#### Description

**Figure 9:**
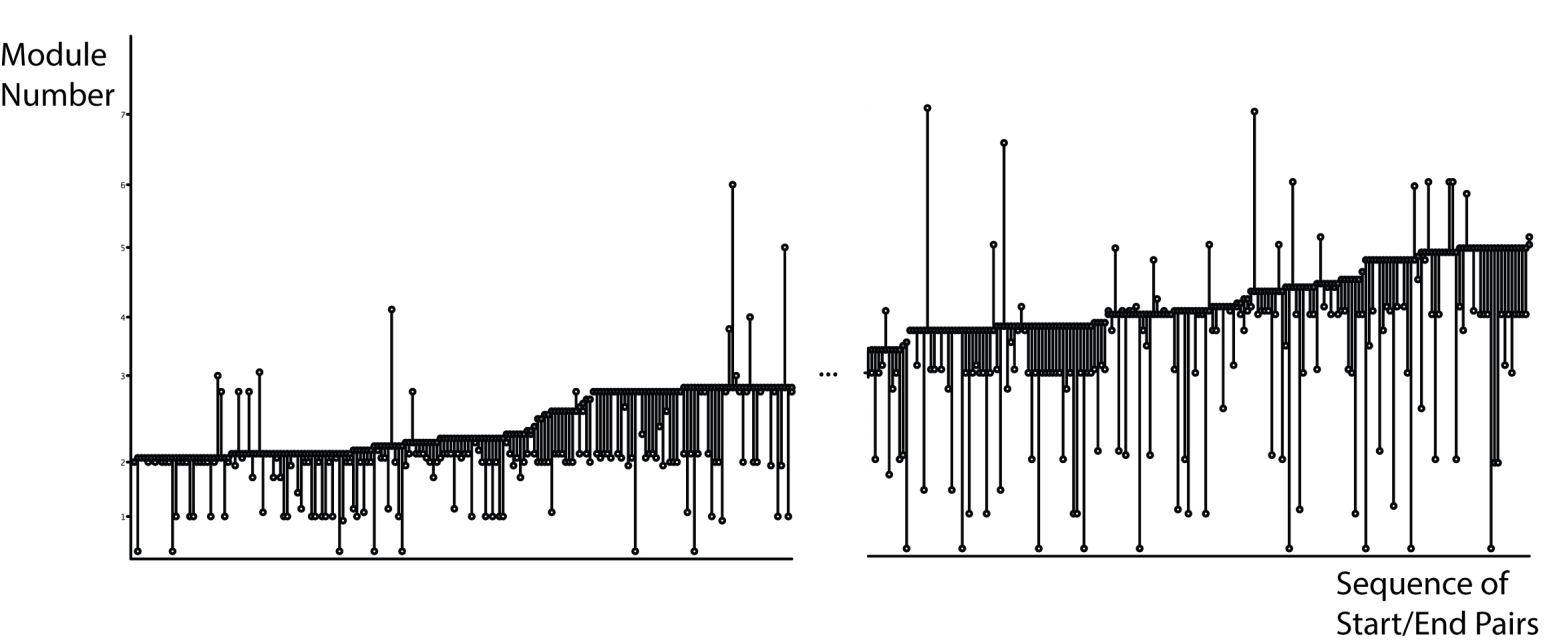
Start-Finish Graph showing plot of start page (light circle) and end page (dark circle) for each session for all users sorted by end page.

The Start–Finish Graph Visualization in Figure 9 plots each user’s starting and finishing content page viewed for every session. It provides an aggregate visualization for session usage of the Web-based intervention. In this case, we have sorted the data points by module number of finishing page visited. The data are excerpted from the large cohort with sections removed to fit the page (the software version can be scrolled).

This visualization captures (1) amount of content covered in individual sessions, (2) direction of content usage, and (3) overlap of module completion.

#### Example

This visualization does not have stepped rows that would suggest people complete one module in a sitting. Instead, we observe that many users did not start at the beginning of one module and stop at the end. Some took several sessions to complete a module, while others did several in one sitting. This is an interesting finding given that many interventions measure their usage through module completion.

**Figure 10:**
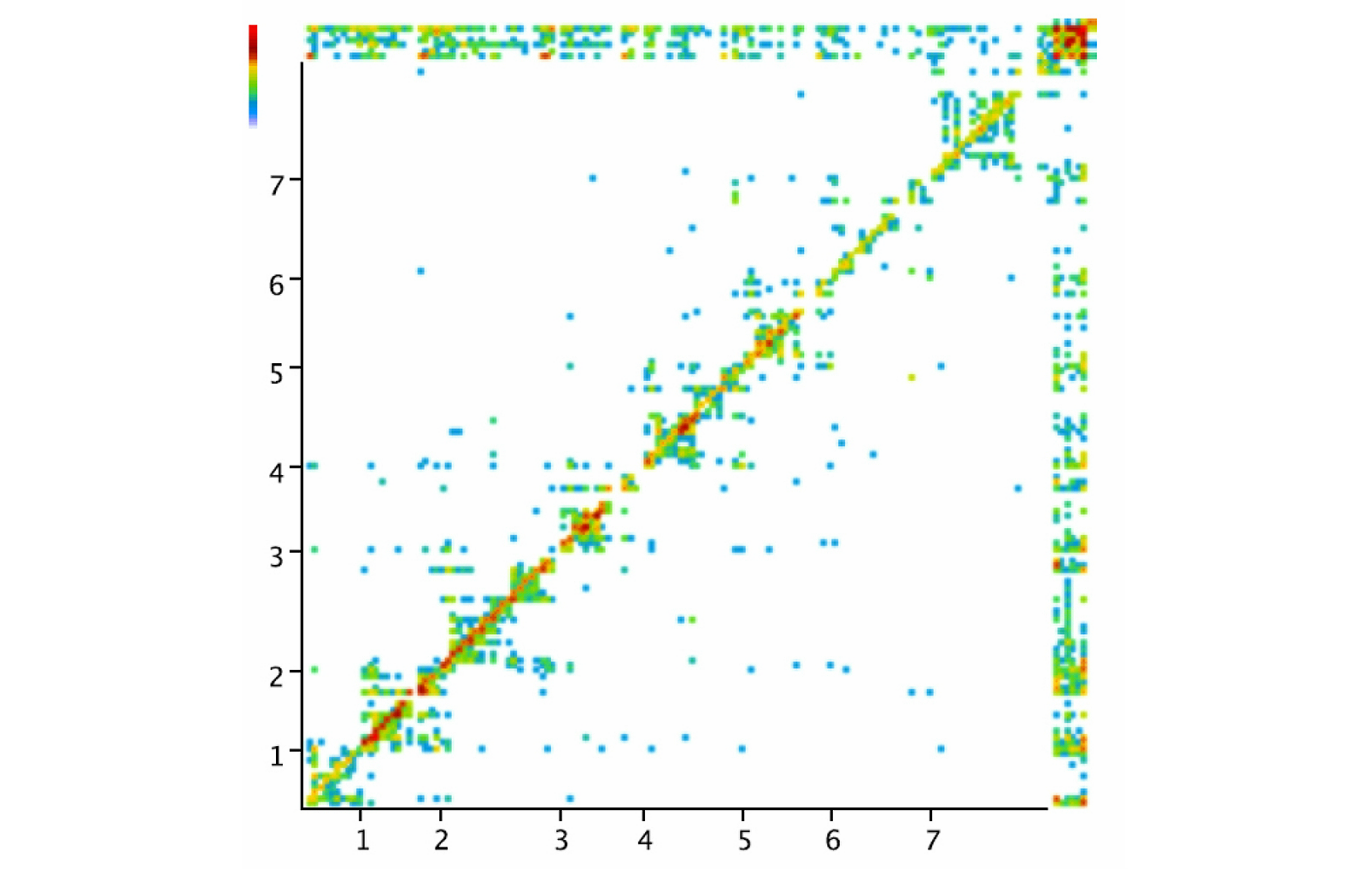
Next Action Heat Map Visualization showing a plot of the likelihood of going from one content page to another: horizontal axis is the module number identifying the content page and the vertical axis the next page view. A red diagonal line would form from the lower left corner if the intervention could only be navigated in a single pre-defined manner.

This visualization can also support the design process by looking for common break points. We can see, for example, that more people seemed to stop at intermediate points between modules 4 and 5 than modules 2 and 3. This finding could prompt a usability evaluation at this point. Taken in conjunction with data in Figures 7, 8, and 10, which suggests that engagement wanes at the end of the intervention, we could also explore different engagement strategies at this point. In this case, the visualization is acting as pointer to further design and evaluation work.

### Next Action Heat Map Visualization

#### Description

The Next Action Heat Map Visualization explores the linearity of Web-based intervention use for an entire cohort. This visualization plots for any given content page the likelihood of going to another content page, creating a heat map. The horizontal axis is the module number identifying the content page and the vertical axis is that of the next page view. If participants were required to navigate the intervention in a single pre-defined manner, then a red diagonal line would form from the lower left corner. We would call this linear use. Places where the data points spread out indicate where users have taken alternative routes. The points across the top and right side are apps usage, showing how they intersperse with content.

This visualization captures (1) linearity of use and (2) potential usability problems.

#### Example

We can observe that in Figure 10, there was the strongest page-by-page usage in module 1, the first content module, with a similar trend to a lesser extent in modules 2 and 3. Many people did not complete module 0—the introduction to the intervention. The review and optional modules, 6 and 7 respectively, were also not done as frequently. The red dots that come at the beginning of modules 1, 2, and 3 in the top row, indicate that people were doing the interactive exercises in the modules consistently. On the other hand, we can see that people were taking advantage of the multiple routes in each module, focusing on content that was of most interest and moving back and forth from apps.

What we find most provocative about this visualization is the demonstration that people used the multiple navigation pathways while still finding the proposed navigation useful. This visualization might have also indicated usability issues apparent with large gaps in the linear navigation. In this case, the gaps corresponded to optional content where multiple different examples were available in a carousel to illustrate the same point (up to 10 examples in some cases). While we did not identify any usability problems for this intervention, this is not surprising given the sustained iterative development process that was carried out before these visualizations were created.

The challenge of this kind of visualization is to organize the raw data in such a way that artefacts do not appear. In this example, we see blue dots (low frequency usage) at random points, jumping from modules 1 to 5 for example. While this may happen on occasion if someone is clicking around trying to re-find something, it can also happen because the Mindfulness app can be reached from a number of locations without any indication in the log-data. At the time of application development, it was not considered that apps accessed from multiple locations should have separate URLs for ease of tracking interaction.

## Discussion

### Principal Results

We have presented four visualizations of user engagement with a Web-based intervention: Navigation Graph, Stripe Graph, Start–Finish Graph, and Next Action Heat Map. These four visualizations provide a novel way to interrogate the patterns of use through log-data of individual usage and specific aspects of cohort usage. We offer these as an alternative view on user engagement with a Web-based intervention than more commonly used summative measures. In this discussion, we articulate how understanding the process of engagement through visualizations can support the design and evaluation of Web-based interventions.

### Design

The visualizations provide examples of how those authoring interventions can inspect the data at different levels of granularity to improve engagement. The Navigation Graph draws attention to strategies that people employed to revisit material. Authors can inspect numerous examples to give them understanding of different ways that material is viewed with in the temporal relationship to the overall intervention. These patterns can then be inspected in the aggregate using the Heat Map Visualization. Alternatively, the intuition afforded by inspecting the data visually can provide the basis for constructing statistical queries on the log-data.

The visualizations of individual patterns of use afford the opportunity to consider personalization in the design process. It is striking how diverse individual patterns of use are in Figures 5-7. This finding indicates that a search for the “best” interactivity may be the wrong line of inquiry. Rather, Web-based interventions need to be designed to enable personalized usage without overwhelming the user with too many options or a lack of direction. It may also be possible to identify different styles of use that could support the tailoring of specific aspects of the intervention. As in the previous example, these visualizations have helped articulate appropriate research questions for pursing better engagement.

The visualization can also help detect usability issues that may mask the underlying effectiveness of the intervention. Both the Start–Finish Graph and the Next Action Heat Map quickly highlight large trends in cohort navigation. If many people are stopping mid-way through a module in the Start–Finish Graph, it suggests that people are losing interest at this point. It may be that the intervention designers need to consider strategies for engagement at this point. Similarly, large breaks in the Next Action Heat Map indicate that many people are following routes other than proposed, highlighting a potential usability or content issue.

Log-data visualization is likely to be most useful in conjunction with other types of data. For example, it can offer a representation to support the elicitation of experience through qualitative interviewing [38] to understand why people used the intervention a particular way. It could also be used as a method of exploratory analysis before the creation and calculation of engagement statistics, a technique currently being developed by Kelders & Gemert-Pijnen [39]. Log-data visualization can, with other methods, add to the richness of understanding engagement needed to support the iterative design approach for complex interventions [40].

### Evaluation

Log-data visualization offers a way to inspect the assumptions of usage embodied in evaluation metrics and criteria. The visualizations presented here question the implicit assumption of linear usage that underpins summative adherence metrics such as intervention completion or module completion. For example, the Stripes Graph shows the adherence is often not consistent but comes in bursts, suggesting that measures of adherence calculated based on weekly usage should not be considered in isolation. The Navigation Graphs and the Start–Finish Graphs both suggest that module completion may not be an accurate reflection of engagement either.

Usage visualizations also offer a different perspective on interactivity. The Navigation Graph, for example, illustrates the back and forth nature of content intake and interactive activities. Comparing different users, we can also see preferences for certain types of interactivity are highly specific to the individual. This view of the data enables a more nuanced conceptualization of engagement, shifting from what content people see, to what content people interact with. This is an important distinction for researchers concerned with the quality of engagement. It also provides a view of interactivity that goes beyond its reduction to individual feature usage.

Log-data visualization also provides new opportunities for evaluation. Summative clinical trials alone are unlikely to provide the flexibility needed to address the continuing ongoing refinement of Web-based interventions. Approaches are required that can adjust to the speed of technology evolution and provide outcome data appropriate to the varied settings and configurations in which a Web-based intervention may be used in practice. Engagement visualizations can allow continual assessment of an intervention as it is changed to incorporate new technologies or adapted to new settings. Visualizations could potentially underpin a more dynamic model of evaluation. Further work would be needed to determine the best visualizations for this purpose.

### Conclusions

The science of engagement is in its infancy. There is much that could be done in the sphere of visualization depending on the size of dataset, granularity of analysis, and motivation for looking at the log-data. Understanding the experience of an individual user (perhaps correlating with qualitative feedback) is at one end of the spectrum, whereas understanding patterns of engagement for a particular demographic would be at the other end. Other uses, such as by clients themselves to reflect upon their own patterns in relationship to the intervention are also possible.

We offer these visualizations as a demonstration of some of the benefits of understanding engagement through log-data. Specifically, we show how visualizations can (1) allow inspection of content or feature usage in a temporal relationship to the overall program at different levels of granularity, (2) detect different patterns of use to consider personalization in the design process, (3) detect usability or content issues, (4) enable exploratory analysis to support the design of statistical queries to summarize the data, (5) provide new opportunities for real-time evaluation, and (6) examine assumptions about interactivity that underlie many summative measures in this field.

We do not suggest that these visualizations are the ultimate set. Indeed, visualization will need to some extent be specific to the design intent of the Web-based intervention as it is best suited to exploratory analysis. Therefore, it is unlikely for the research community to settle on a single set of visualizations for all interventions, but we felt that having a starting point would be useful. We have made the programs freely available to encourage others to explore these visualizations with their own data.

A shared toolset within the community, which allows exploratory and not just confirmatory analysis of data, will require some degree of standardization, for example through a common format for log-data from online interventions. Common fields such as event identifier (URL), timestamp, and user identifier are clearly required, other data points might also be considered, such as self-report measures and treatment events (eg, support sessions for clients receiving blended treatment). The model presented in Figure 1 illustrates one possible arrangement of such data. This would allow researchers developing different interventions to share a common set of tools for visualizing and analyzing log-data.

Future research in this area could help to provide the rigor of development for engagement currently sought to address the issues of adherence that stymie the regular use of Web-based interventions.

## Multimedia Appendix 1

Processing code and test data for the four visualizations.

## Abbreviations

NHS: National Health Service
NIHR: National Institute for Health Research
URL: uniform resource locator
