# Supplementary material for: Analyzing Engagement in a Web-Based Intervention Platform Through Visualizing Log-Data
Source: J Med Internet Res. 2014 Nov 13;16(11):e252. doi: 10.2196/jmir.3575 (PMC4260085; doi:10.2196/jmir.3575)
Supplement: Supplementary file 1 [file jmir_v16i11e252_app1.zip › Submission_files/graph_next_action/web-export/index.html]

finish\_point : Built with Processing and Processing.js


Your browser does not support the canvas tag.


JavaScript is required to view the contents of this page.

# finish\_point

Source code: finish\_point

Built with Processing
and Processing.js
