# Supplementary figures and images for: Analyzing Engagement in a Web-Based Intervention Platform Through Visualizing Log-Data
Source: J Med Internet Res. 2014 Nov 13;16(11):e252. doi: 10.2196/jmir.3575 (PMC4260085; doi:10.2196/jmir.3575)

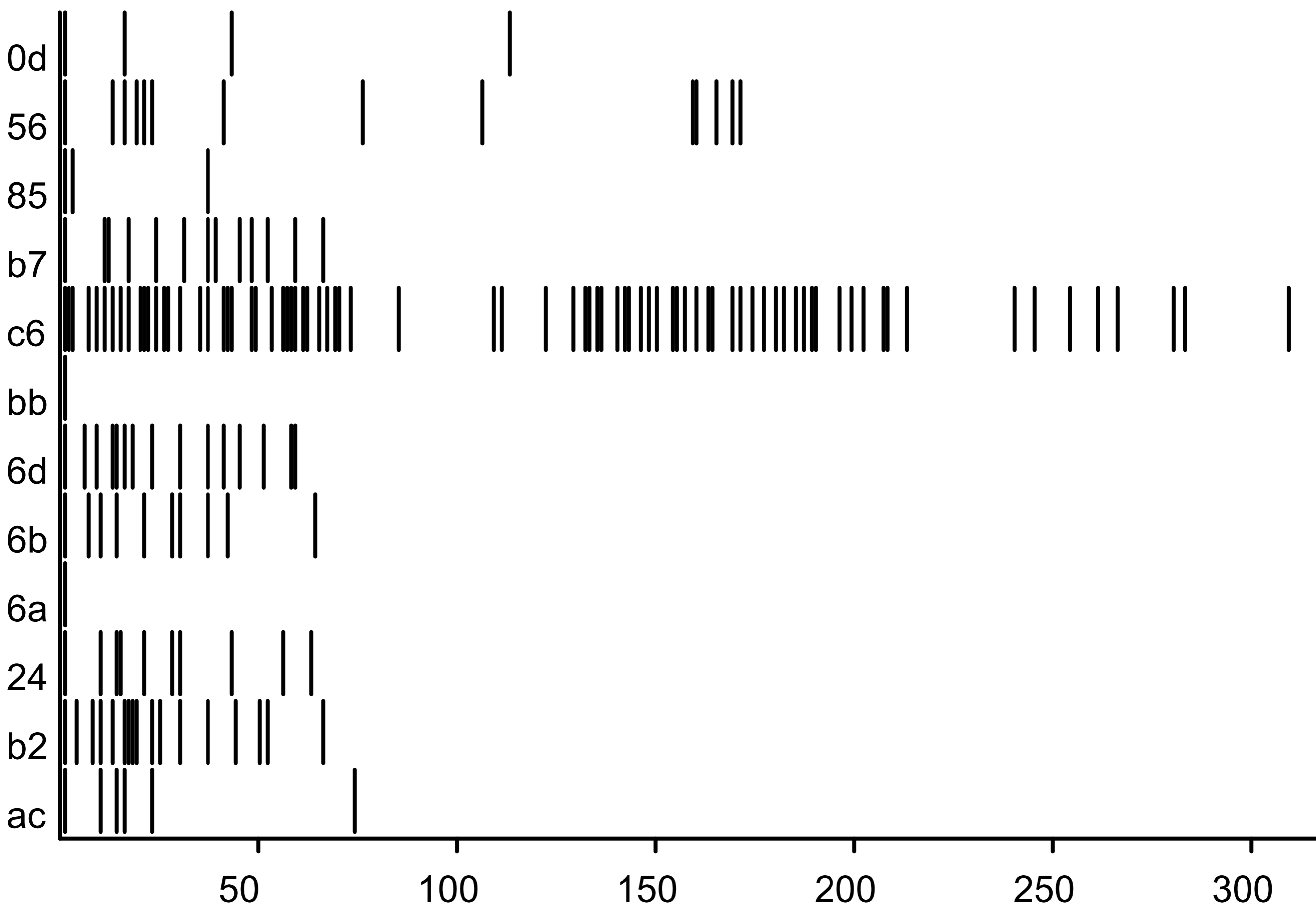

Supplement: Supplementary file 1 [file jmir_v16i11e252_app1.zip › Submission_files/graph_days_stripe/output0.pdf]

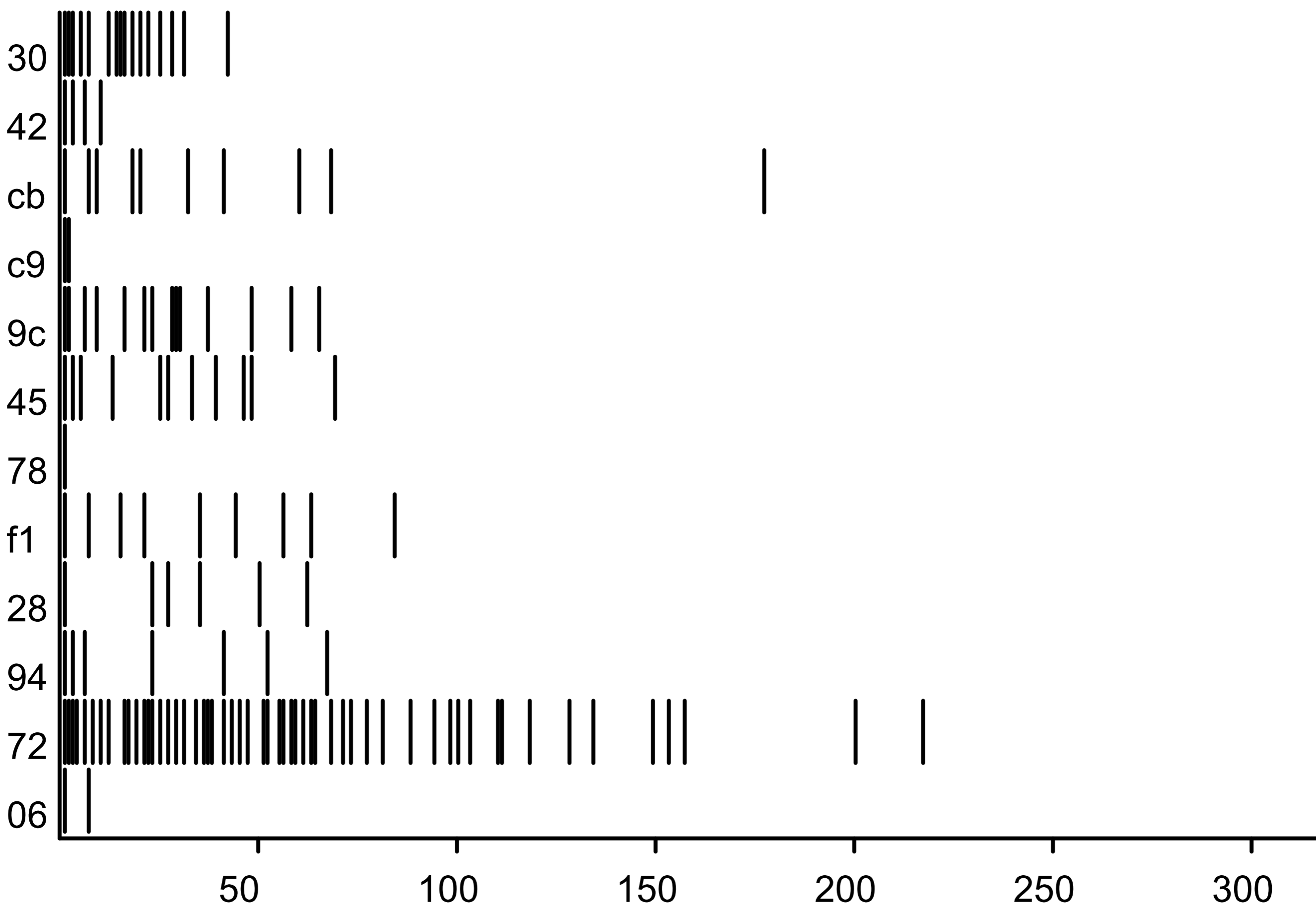

Supplement: Supplementary file 1 [file jmir_v16i11e252_app1.zip › Submission_files/graph_days_stripe/output1.pdf]

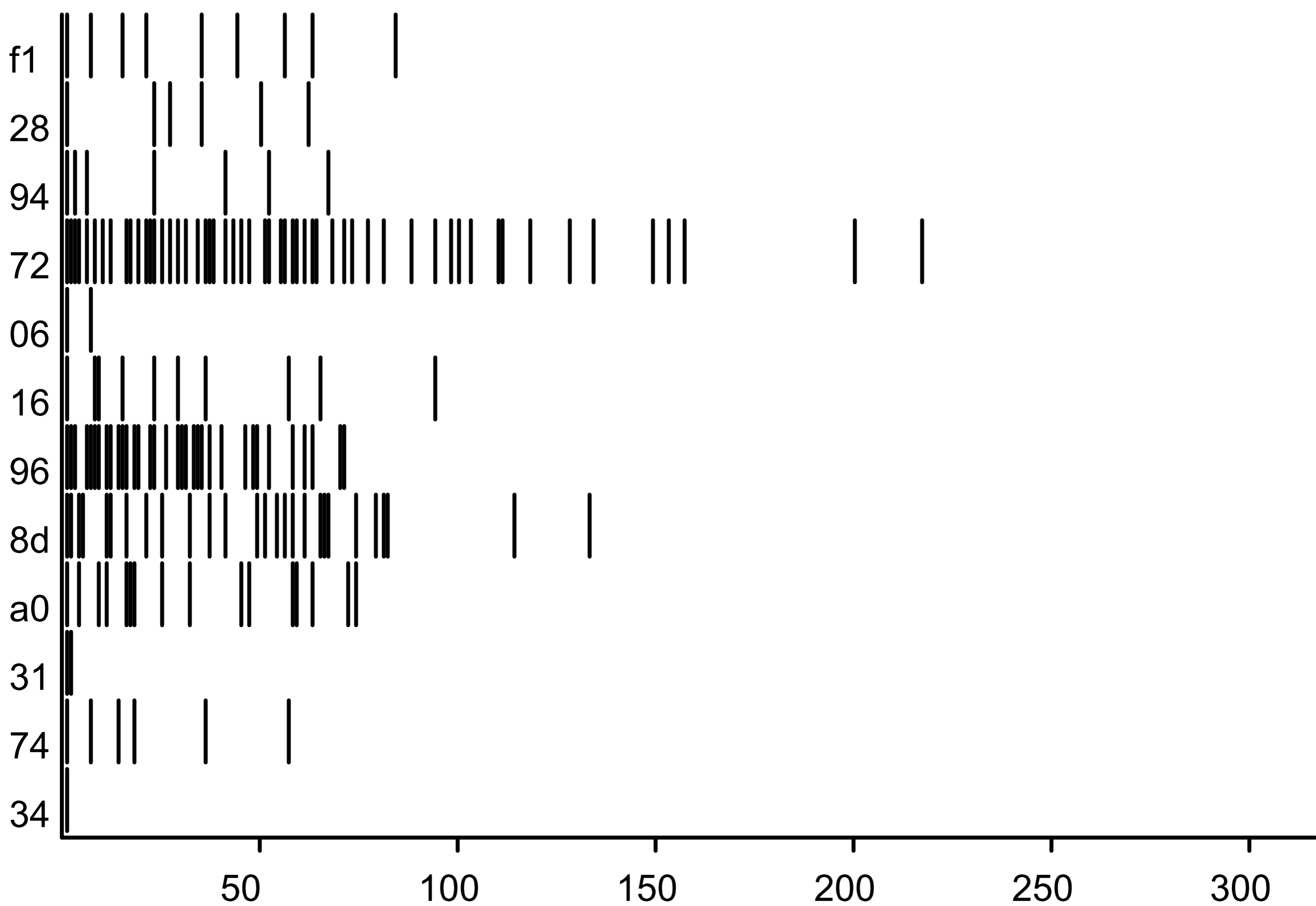

Supplement: Supplementary file 1 [file jmir_v16i11e252_app1.zip › Submission_files/graph_days_stripe/output2.pdf]

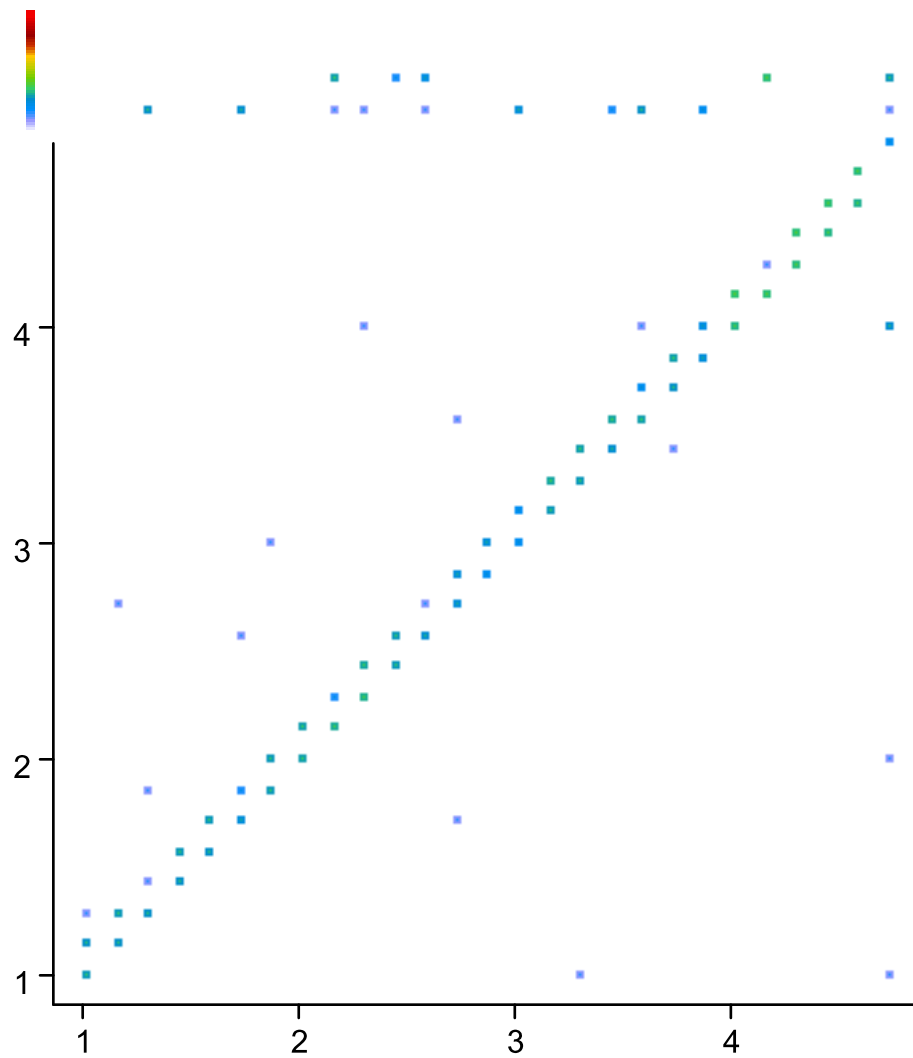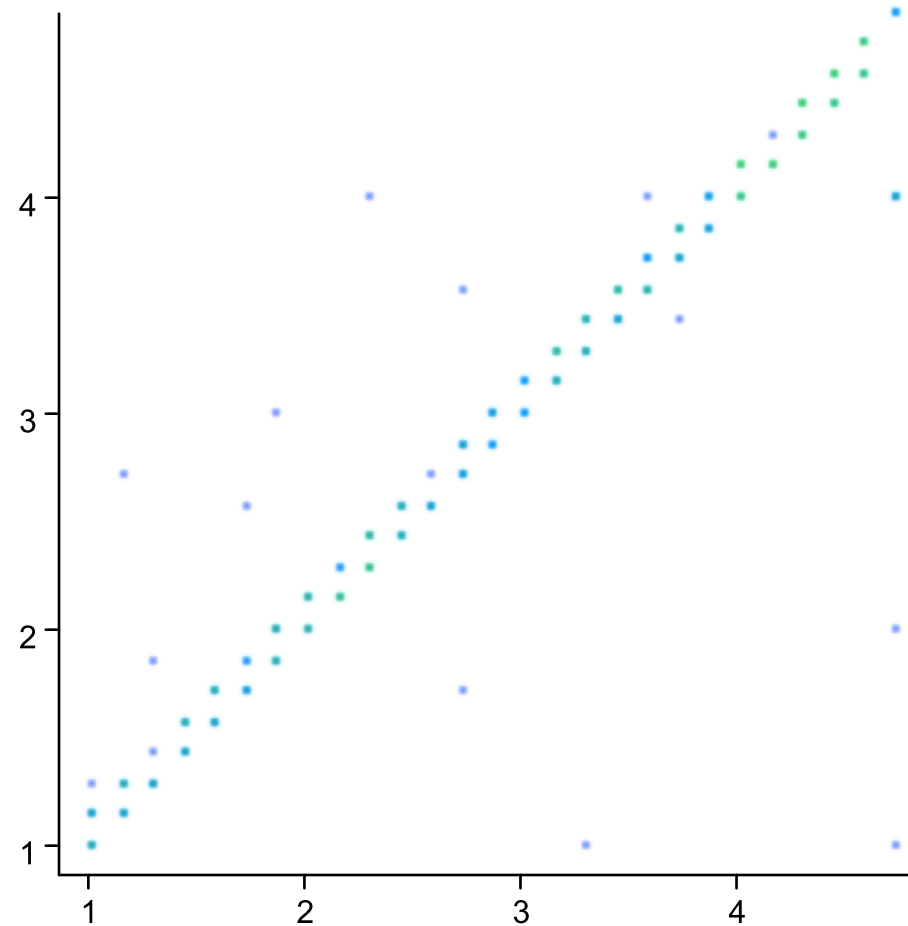

Supplement: Supplementary file 1 [file jmir_v16i11e252_app1.zip › Submission_files/graph_next_action/output.pdf]
